# Revisiting the tryptophan-serotonin deficiency and the inflammatory hypotheses of major depression in a biopsychosocial approach

**DOI:** 10.7717/peerj.3968

**Published:** 2017-11-02

**Authors:** Andreas Baranyi, Omid Amouzadeh-Ghadikolai, Dirk von Lewinski, Robert J. Breitenecker, Hans-Bernd Rothenhäusler, Christoph Robier, Maria Baranyi, Simon Theokas, Andreas Meinitzer

**Affiliations:** 1 Department of Psychiatry and Psychotherapeutic Medicine, Medical University of Graz, Graz, Austria; 2 Hospital of the Brothers of St. John of God, Graz, Austria; 3 Division of Cardiology, Department of Internal Medicine, Medical University of Graz, Graz, Austria; 4 Institute for Innovation, Johannes Kepler University, Linz, Austria; 5 Clinical Institute of Medical and Chemical Laboratory Diagnostics, Medical University of Graz, Graz, Austria

**Keywords:** Major depression, Tryptophan-serotonin deficiency hypothesis, Inflammatory hypothesis, Life satisfaction, Social support, Health-related quality of life

## Abstract

**Background:**

The aim of this cross-sectional study was to identify important biopsychosocial correlates of major depression. Biological mechanisms, including the inflammatory and the tryptophan-serotonin deficiency hypotheses of major depression, were investigated alongside health-related quality of life, life satisfaction, and social support.

**Methods:**

The concentrations of plasma tryptophan, plasma kynurenine, plasma kynurenic acid, serum quinolinic acid, and the tryptophan breakdown to kynurenine were determined alongside health-related quality of life (Medical Outcome Study Form, SF-36), life satisfaction (Life Satisfaction Questionnaire, FLZ), and social support (Social Support Survey, SSS) in 71 depressive patients at the time of their in-patient admittance and 48 healthy controls.

**Results:**

Corresponding with the inflammatory hypothesis of major depression, our study results suggest a tryptophan breakdown to kynurenine in patients with major depression, and depressive patients had a lower concentration of neuroprotective kynurenic acid in comparison to the healthy controls (Mann–Whitney-U: 1315.0; *p* = 0.046). Contradicting the inflammatory theory, the concentrations of kynurenine (*t*: −0.945; *df* = 116; *p* = 0.347) and quinolinic acid (Mann-Whitney-U: 1376.5; *p* = 0.076) in depressive patients were not significantly different between depressed and healthy controls. Our findings tend to support the tryptophan-serotonin deficiency hypothesis of major depression, as the deficiency of the serotonin precursor tryptophan in depressive patients (*t*: −3.931; *df* = 116; *p* < 0.001) suggests dysfunction of serotonin neurotransmission. A two-step hierarchical linear regression model showed that low tryptophan concentrations, low social support (SSS), occupational requirements (FLZ), personality traits (FLZ), impaired physical role (SF-36), and impaired vitality (SF-36) predict higher Beck Depression Inventory (BDI-II) scores.

**Discussion:**

Our study results argue for the validity of a biopsychosocial model of major depression with multiple pathophysiological mechanisms involved.

## Introduction

Traditional biomedical models of clinical medicine focus on pathophysiology and biological approaches to illness. As an extension, the biopsychosocial model gives priority to understanding health and illness in their full contexts, systematically including the main contributing biological and psychosocial factors and their complex interactions (Engel, 1977). The etiology and biological causes of major depression so far have not been fully identified (Kasper, 2010; Kasper et al., 2012). In addition, there is a high variability in the structural changes (e.g., hippocampal atrophy) in patients with major depression, and appropriate biomarkers of major depression have not yet been identified (Bijanki et al., 2014). Biological mechanisms apply in several hypotheses, including the Tryptophan-Serotonin (TRP-5-HT) deficiency hypothesis as well as the inflammatory and neurodegeneration hypothesis of major depression (Bijanki et al., 2014; Patten, 2015).

### Tryptophan-serotonin (TRP-5-HT) deficiency hypothesis of depression

The TRP-5-HT deficiency hypothesis of major depression focuses on the dysfunction of Serotonin (5-HT) neurotransmission in the pathophysiology of patients with major depression (Fava & Sonino, 2010). In addition, patients with major depression have low levels of plasma L- Tryptophan (TRP) available for their 5-HT synthesis. Physiological changes in plasma TRP concentrations might impact on brain 5-HT levels, due to the ability of TRP to cross the blood-brain barrier (BBB) via a cerebral transport system, which includes the competing large neutral amino acids (Fernstrom & Wurtman, 1971; Wichers et al., 2005).

### Inflammatory and neurodegeneration hypothesis of depression

A common finding both in chronic inflammation and during infectious diseases is sickness behavior. Sickness behavior includes a set of behavioral changes: lack of movement, indifference, self-neglect and reduced intake of food, even though an organism in a state of fever is commonly metabolically more active (Dantzer & Kelley, 2007). In the past, sickness behavior was thought to be a sign of unspecified physical weakness of the organism, while today sickness behavior is more understood to be an immunologically mediated strategic adjustment that aims to protect the organism in order to provide energy for coping with sickness (Kelley et al., 2003). Sickness behavior is triggered by pro-inflammatory cytokines. In animal studies with rats, intracerebral injection of TH1 cytokines (mainly Interferon [IFN]-α) resulted in depressed mood, dysphoria, anhedonia, anxiety, exhaustion, fatigue, psychomotor retardation, insomnia, cognitive dysfunction (memory, lack of concentration), pain, weight loss due to lack of appetite, and fever (Dantzer & Kelley, 2007). Many of these symptoms are similar to symptoms shown by depressive patients. This observation has led to the development of the inflammatory and neurodegeneration hypothesis of major depression (Kelley et al., 2003).

It is a well-known fact that major depression is often accompanied by changes in immune functions (Steiner et al., 2011). There is a plethora of immunological consequences, and so far, the current state of research has not produced one consistent overview (Steiner et al., 2012). However, a neuro-inflammatory response (e.g., increase of monocyte-derived cytokines such as Interleukin (Il)-1, Il-6, TNF-α), a decrease in activity of natural killer cells and an increased concentration of microglia in specific areas of the brain are detected in a large number of patients suffering from major depression. (Steiner et al., 2011; Steiner et al., 2012). Significantly higher concentrations of TNF-α and IL-6 in depressed subjects compared with control subjects have also been determined by means of a large meta-analysis of studies measuring cytokine concentration in patients with major depression (Dowlati et al., 2010). Another recent systematic review and meta-analysis of studies that measured cytokine and chemokine levels in patients with major depression compared to healthy controls revealed that peripheral levels of IL-6, TNF-α, IL-10, the soluble IL-2 receptor, C-C chemokine ligand 2, IL-13, IL-18, IL-12, the IL-1 receptor antagonist, and the soluble TNF receptor 2 are elevated in patients with major depression compared to healthy controls. In this meta-analysis IFN-*γ* levels were lower in patients with major depression (Köhler et al., 2017).

In the context of the inflammatory and neurodegeneration hypothesis of major depression, depressive illness is seen as a psychopathological manifestation of central inflammatory processes (Steiner et al., 2011; Steiner et al., 2012). Thus, TNF-α, FN-α- *α* and IFN-*γ* have a substantial influence on Indoleamine 2,3-dioxygenase (IDO) 1 activity. IDO1 is expressed in a number of cell types, including microglia, dendritic cells, fibroblasts and monocytes. In addition to Tryptophan 2,3-dioxygenase (TDO) and to the previously mentioned classic IDO1 (previously known as IDO), a new variant, IDO2, has been described (Löb et al., 2009). IDO1 is a TRP-catabolizing enzyme that causes a greater breakdown of TRP to Kynurenine (KYN). KYN is able to cross the blood- brain barrier, and is subsequently broken up into its neurotoxic metabolites by human microglia (Wichers et al., 2005). An important neurotoxic KYN metabolite is Quinolinic Acid (QA), which might trigger depressive symptoms. Thus, QA is a potent N-methyl-D-aspartate (NMDA) receptor agonist and activates the NMDA receptor in pathophysiological concentrations, and a massive calcium entry into neurons is the consequence. Especially neurons located in areas that contain a particularly high number of NMDA receptors (e.g., hippocampus, striatum and neocortex) are extremely sensitive to QA (Vandresen-Filho et al., 2015; Schwarcz & Köhler, 1983). Furthermore, QA can cause greater glutamate release by neurons and inhibits its reuptake by astrocytes. Excessive microenvironment glutamate concentrations cause neurotoxicity (Wichers & Maes, 2002; Wichers et al., 2005; Tavares et al., 2002). Beta-Trace Protein (BTP) might be a non-invasive biomarker to indicate QA-induced impaired blood- brain barrier integrity (Baranyi et al., 2017). Another metabolite of KYN is Kynurenic Acid (KA), an antagonist of the glutamate recognition site of the NMDA receptor. In this way, KA may prevent NMDA overstimulation. However, KYN aminotransferase, an enzyme that synthesizes KA, is inferior to the direct pathway from KYN to QA, as IDO1 stimulates kynureninase and additionally hinders the activity of KYN aminotransferase (Wichers et al., 2005).

### Psychosocial factors

A comprehensive biopsychosocial model of major depression should include not only potential biological mechanisms for depressive disorders but also psychosocial factors, including health-related quality of life, life satisfaction, social influences and major life stressors (Fava et al., 2012; Baranyi et al., 2013; Baranyi, 2014; Baranyi et al., 2015a; Baranyi et al., 2015b). Thus, major life stressors, especially those involving interpersonal stress and social rejection, are important risk factors for depression (Slavich & Irwin, 2014). The well-validated social signal transduction theory of major depression characterizes important physiologic, neural, molecular, and genomic mechanisms that link experiences of social-environmental stress with internal biological processes that might be important for pathogenesis of major depression (Slavich & Irwin, 2014). In this theory, high social threat or adversity up-regulates inflammation processes of the immune system. The key immune response mediators are the proinflammatory cytokines, which in turn lead to behaviour changes (e.g., depressive symptoms, anhedonia, or psychomotor retardation; Slavich & Irwin, 2014). Corresponding with the concept of the transduction theory Pace et al. (2006) showed in an earlier study that male patients with major depression and a history of increased early life stress had enhanced psychosocial stress-induced increases in IL-6 and NF-kappaB DNA-binding.

### Aims of the study

Regarding major depression, previous research has tended to pay attention either to pathophysiological pathways or to important contributing psychosocial factors. Thus, the synopsis of all contributing factors into one holistic biopsychosocial model is a main scientific goal (Fava et al., 2012; Baranyi et al., 2013; Baranyi, 2014), and the prior aim of this cross-sectional exploratory study was to identify important biopsychosocial correlates of major depression. Biological mechanisms, including the inflammatory hypothesis and the TRP-5-HT deficiency hypothesis of depression, were investigated alongside health-related quality of life, life satisfaction and social support, and patients with major depression were directly compared with healthy controls.

## Materials & Methods

### Participants

This study is part of our research project on the ethiopathogenesis and health consequences of major depression. In former published studies (Baranyi et al., 2015a; Baranyi et al., 2016; Baranyi et al., 2017) of this research project we have investigated primarily the impact of beta-trace protein (BTP) as a new non-invasive immunological marker for QA-induced impaired blood-brain barrier integrity (Baranyi et al., 2017). Later we have described the nitric oxide-related biological pathways (Baranyi et al., 2015a), and finally we have explored the impact of branched-chain amino acids (BCAAs) as new biomarkers of major depression (Baranyi et al., 2016). A total of 122 subjects (74 patients with major depression at the time of in-patient admittance to the Department of Psychiatry, Hospital of the Brothers of St. John of God, Graz, Austria, and 48 somatically healthy controls without current or former depression and without any former history of psychiatric disorders) were initially recruited for this ongoing research project. Healthy controls were recruited using flyers that briefly introduced the research project and provided contact details of the study’s staff. Three depressive participants then refused to participate in the study after signing the informed consent form. Thus, the final study sample consisted of 71 patients with major depression and the 48 anamnestic somatically healthy controls without current or former depression and without any former history of psychiatric disorders.

The subsequent reasons for exclusion from enrolment applied to all subjects (patients with major depression and healthy controls): (1) pregnancy, (2) clinically significant co-morbid conditions (e.g., anamnestic significant cardio-vascular illness, cancer), (3) disease or drugs that might influence the immune system, (4) signs of infection and (5) anamnestic diagnosis of a neurological disease.

This research project has been approved by the Institutional Review Board of the Medical University of Graz (IRB approval number EK-17-031 ex 05/06). Data protection met the standards set by Austrian law. The methods were carried out in accordance with the approved guidelines. All participants in this study had to give signed informed consent, and subjects could decide to withdraw from this research project at any time.

### Biological assessments

Blood was sampled from the fasting subjects with major depression between 08.00 and 09.00 am at the time of in-patient admittance to the Department of Psychiatry for the assays of TRP, KYN, KA and QA. The already mentioned first study (Baranyi et al., 2017) of our research project of the impact of BTP as a marker for QA-induced impaired blood-brain barrier integrity consisted of a smaller data set of only 61 patients with major depression and 45 healthy controls. Thus, a part of the QA data of this enlarged present study has already been published in a completely different context, where it was evaluated for the first time whether BTP might be a new non-invasive immunological marker for QA-induced impaired blood-brain barrier integrity (Baranyi et al., 2017).

For an estimation of TRP breakdown to KYN (due to either activated IDO1, IDO2 or TDO), a Pearson correlation coefficient between TRP and KYN, and additionally the KYN to TRP quotient, was computed. The same biological assessments were carried out in the healthy controls without depressive symptomatology. From each subject, 16.5 mL of blood were drawn after short tourniquet using a 21-gauge needle into serum- (7.5 mL) and EDTA- (9 mL) sample tubes (Sarstedt, Nuembrecht, Germany). Prior to centrifugation, the serum tubes were kept in a vertical position at room temperature until blood coagulation had completed. TRP, KYN and KA were measured in plasma samples by HPLC with a simultaneous ultraviolet and fluorometric detection system (Hervé et al., 1996; Xiao et al., 2008; US Department of Health and Human Services et al., 2001).

Our research team previously established a new and validated liquid chromatography-tandem mass spectrometric method for the determination of QA, which is described in detail elsewhere (Meinitzer et al., 2014). QA was measured in frozen serum. Within-day coefficients of variation and between-day coefficients of variation were all below 10%.

### Psychometric assessments

All consenting subjects were interviewed by experienced consultant-liaison psychiatrists (A.B., O.A.-G). Every subject completed a research battery consisting of a brief author-compiled questionnaire on sociodemographic characteristics and the self-reporting questionnaires Beck Depression Inventory (BDI) -II (Beck et al., 1961), the Medical Outcome Study Form (SF-36; Stewart, Hays & Ware, 1988), the Life Satisfaction Questionnaire (FLZ; Fahrenberg, Myrtek & Schuhmacher, 2000), and the Social Support Survey (SSS; Sherbourne & Stewart, 1991).

### Sociodemographic characteristics and psychometric assessments

#### Sociodemographic characteristics

An author-compiled self-rating questionnaire was used to obtain information about the main ociodemographic characteristics of the subjects (e.g., age, gender, employment, living arrangements, and marital status at the time of psychiatric assessment).

#### Beck Depression Inventory (BDI) -II (Beck et al., 1961; Hautzinger, Keller & Kühner, 2009)

The BDI-II is a 21-question multiple-choice survey widely used for measuring the severity of depressive symptomatology. The self-rating questionnaire consists of 21 items relating to depressive symptomatology (Beck et al., 1961; Hautzinger, Keller & Kühner, 2009).

#### Medical outcome study form (SF-36; Stewart, Hays & Ware, 1988)

To assess health-related quality of life, we applied the psychometrically well-validated and widely used SF-36, a 36-item self-rating questionnaire that covers eight health-related domains: Physical Functioning, Physical Role, Body Pain, General Health, Vitality, Social Functioning, Emotional Role, and Mental Health. Each of these 8 domains yields a score ranging from 0 (worst) to 100 (best). In the vast majority of the already published studies, the internal-consistency data of the SF-36 exceed 0.8 (Stewart, Hays & Ware, 1988; Ware et al., 2008).

#### Life satisfaction questionnaire (FLZ; Fahrenberg, Myrtek & Schuhmacher, 2000)

To assess life satisfaction, we applied the psychometrically well-validated self-rating Life Satisfaction Questionnaire (FLZ) that covers the main life satisfaction domains: General Health; Occupational Requirements; Income/Financial Security; Leisure Time/Hobbies; Partner Relationship; Family Life/Children; Personality Traits; Sexuality; Friends/Acquaintances; Housing/Living Conditions. Each of these domains covers seven items that are rated on a seven-point scale ranging from “very unsatisfied” to “very satisfied”. The internal-consistency data of the FLZ are between 0.82 and 0.95 (Fahrenberg, Myrtek & Schuhmacher, 2000).

#### Social Support Survey (SSS; Sherbourne & Stewart, 1991)

The SSS is a multidimensional self-report questionnaire of perceived social support. The 19 items have a rating scale from 0 (never) to 4 (always). Higher scores reflect a more positive perception of social support (Sherbourne & Stewart, 1991; Rees et al., 2000).

### Statistical analyses

Descriptive statistics were produced based on sociodemographic, biochemical and psychometric data, and are presented as mean, standard deviation (SD), median and range. We applied *χ*^2^-tests to evaluate group differences concerning patients with major depression and healthy controls in categorical sociodemographic variables. For continuous measures, differences between groups were assessed using the Mann–Whitney *U* test, or, where appropriate, *t*-tests. To correct for multiple comparisons in the cases of the Mann–Whitney *U* test and *t*-tests of the biochemical and psychometric assessments we applied the Benjamini–Hochberg procedure based on a false discovery rate of 0.1 (Hochberg & Benjamini, 1990; McDonald, 2014). Further, to discover important correlates of depression (BDI-II scores) we selected potential sociodemographic, biochemical and psychometric variables. To avoid multi-collinearity in the further regression analysis we selected appropriate biochemical measures based on bivariate correlations (Spearman rank correlations and Partial Correlation coefficients). Then, we estimated a two-step hierarchical linear regression model. We started with a model including control variables. Further, we entered all selected biological and psychometric predictor variables into the regression model. All statistical analyses were performed with IBM-SPSS Statistics 23.0 for Windows.

## Results

### Sociodemographic, clinical and treatment characteristics

The basic sociodemographic, clinical (BDI-II) and treatment characteristics of the 71 depressive patients and the 48 healthy controls have already been reported in detail in previously published studies about nitric oxide-related biological pathways and about the impact of BCAAs as new biomarkers of major depression (Baranyi et al., 2016). Nevertheless, for an easier interpretation of the new data they are also repeated in this paper. All subjects were Caucasian. At the time of in-patient admittance to the Department of Psychiatry the depressive patients had a mean BDI-II score of 24.5 (SD = 9.7) and the healthy controls had a mean BDI-II score of 1.52 (SD = 1.92) at the time of their psychiatric assessment. The healthy controls had no history of any previous mental disease. There were no significant sociodemographic differences between the study participants suffering from major depression and the healthy controls regarding their sex, age, marital status, employment status and living arrangements. However, the depressive patients had a significantly higher Body Mass Index (BMI). Most of the depressive study participants had an antidepressant medication treatment at the time of the psychiatric assessment in this study (29 patients (40.8%) had an antidepressant treatment with selective serotonin re-uptake inhibitors (SSRIs), 22 patients (31.0%) had an antidepressant treatment based on serotonin–norepinephrine reuptake inhibitors (SNRIs), eight patients (11.2%) had a prescription of other antidepressants (e.g., bupropion), and 12 (16.9%) depressive patients had no antidepressants prescribed at the time of their study participation).

Table 1 summarizes the sociodemographic characteristics of all subjects and Table 2 the BDI-II scores for the participants with major depression and the healthy controls.

**Table 1:** Sociodemographic Characteristics (Baranyi et al., 2015a; Baranyi et al., 2015b; Baranyi et al., 2016).

Category	Patients with major depression (*n* = 71)	Healthy controls (*n* = 48)	Test for differences
**Sex**			
Male	47 (66.2%)	31 (64.6%)	*χ*^2=^0.033; df = 1; *p* = 0.856^a^
Female	24 (33.8%)	17 (35.4%)	
**Age**			
Mean (years)	49.15	46.06	Mann–Whitney-U: 1985.0; *p* = 0.13^b^
SD	11.4	18.3	
Median	50.3	40.4	
Range	56	55	
**Body Mass Index (BMI)**			
Mean	28.44	24.83	Mann–Whitney-U: 1950.5; *p* < 0.001^b^
SD	7.0	5.2	
Median	26.2	22.9	
Range	38.2	23.2	
**Marital status**			
Single	16 (23.2%)	17 (35.4%)	*χ*^2=^2.608; df = 3; *p* = 0.456^a^
Partner	48 (69.6%)	28 (58.3%)	
Widowed	2 (2.9%)	2 (4.2%)	
Divorced	3 (4.3%)	1 (2.1%)	
**Employment status**			
Paid work	32 (46.4%)	23 (47.9%)	*χ*^2=^0.27; df = 1; *p* = 0.870^a^
No paid work (homeworker, un-employed, retired)	37 (53.6%)	25 (52.1%)	
***Living arrangements***			
Alone	18 (26.1%)	11 (22.9%)	*χ*^2=^0.153; df = 1; *p* = 0.696^a^
With others (family, partner or friends)	51 (73.9%)	37 (77.1%)	

**Notes.**

Legend SD Standard deviation

a *χ*^2^ tests.

b Mann–Whitney- *U*-test.

**Table 2:** Biological Assessments and BDI-II scores for the patients with major depression at the time of in-patient admittance and healthy controls.

	Patients with major depression (*n* = 71)	Healthy controls (*n* = 48)		Test for differences
BDI-II	Mean: 24.5 SD: 9.7 Median: 24.0 Range: 39	Mean: 1.5 SD: 1.9 Median:1.0 Range: 8	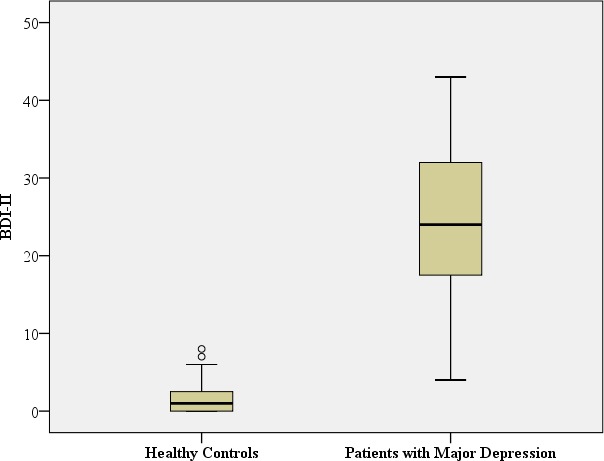	Mann–Whitney-U: 9.5; ***p* < 0.001**^a^ Benjamini Hochberg Critical Value^c^for multiple comparisons: 0.017^*^
**TRP,** µmol/L	Mean: 60.1 SD: 10.88	Mean: 67.7 SD: 9.50	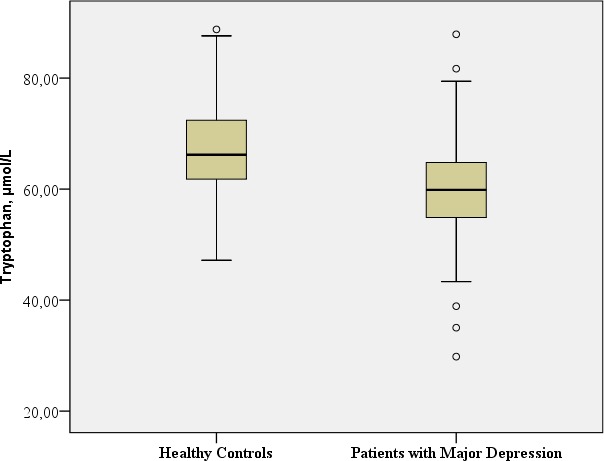	t: −3.931; df = 116; ***p*< 0.001**^a^ Benjamini Hochberg Critical Value^c^ for multiple comparisons: 0.033^*^
**KYN,** µmol/L	Mean: 2.4 SD: 0.62	Mean: 2.5 SD: 0.62	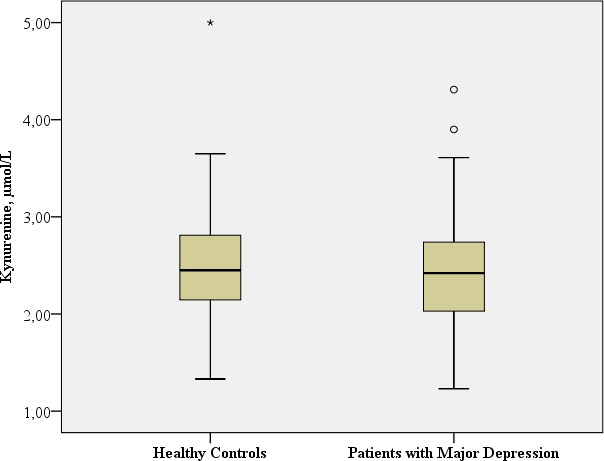	t: −0.945; df = 116; *p* = 0.347^a^ Benjamini Hochberg Critical Value^c^ for multiple comparisons: 0.1^*^
**TRP breakdown to KYN** (KYN/TRP Ratio) **Spearman rank correlation coefficients between KYN and TRP**	Mean: 0.0409 SD: 0.011 Median: 0.040 Range: 0.055 **Spearman-Rho:***r* = 0.253, *p* = 0.035	Mean: 0.0380 SD: 0.011 Median: 0.035 Range: 0.0628 **Spearman-Rho:***r* = − 0.005, *p* = 0.975	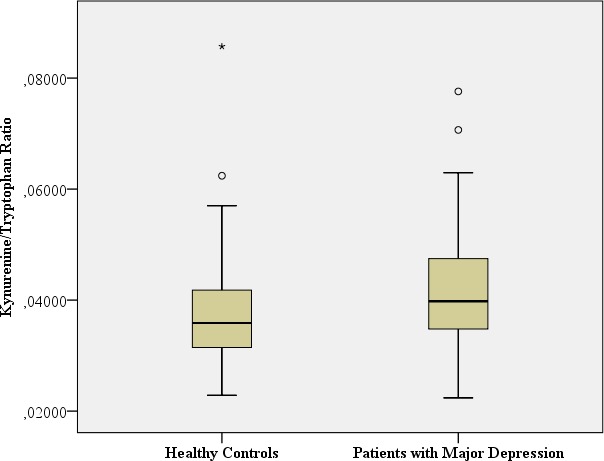	Mann–Whitney-U: 1338.0; *p* = 0.061^a^ Benjamini Hochberg Critical Value^c^ for multiple comparisons: 0.067^*^
**QA,** nmol/L	Mean: 370.57 SD: 126.12 Median: 348.80 Range: 544.20	Mean: 428.11 SD: 194.96 Median: 390.1 Range: 1049.5	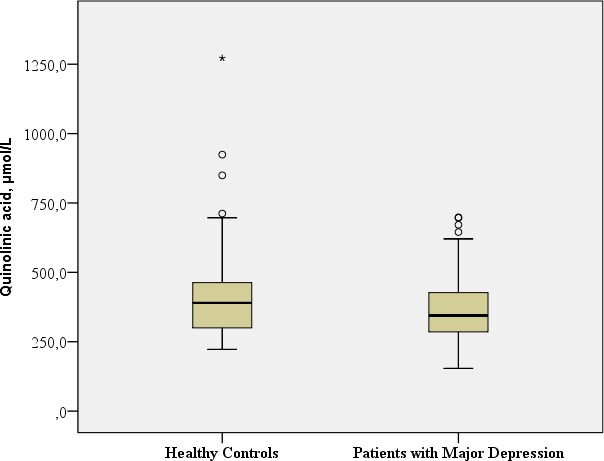	Mann–Whitney-U: 1376.5; *p* = 0.076^a^ Benjamini Hochberg Critical Value^c^ for multiple comparisons: 0.083^*^
**KA,** nmol/L	Mean: 34.54 SD: 26.08 Median: 30.74 Range: 213.05	Mean: 37.40 SD: 14.61 Median: 34.08 Range: 67.51	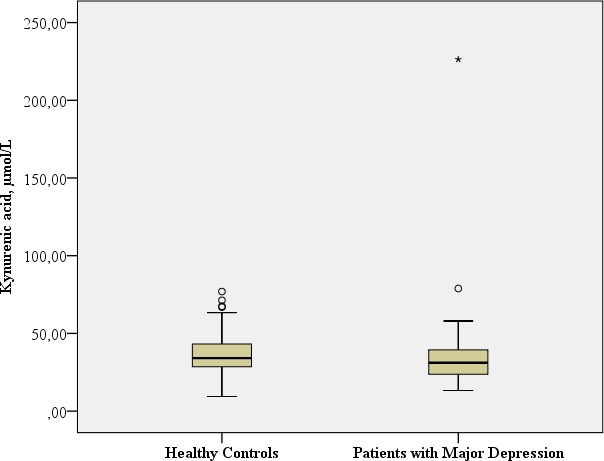	Mann–Whitney-U: 1315.0; *p* = 0.046^a^ Benjamini Hochberg Critical Value^c^ for multiple comparisons: 0.05^d^

**Notes**

a Mann–Whitney-U-test.

b *t*-test.

c Benjamini Hochberg critical value: (i/m)xQ; False Discovery Rate: 0.1.

d Test significant concerning Benjamin-Hochberg correction.

### TRP-5-HT deficiency hypothesis and inflammatory hypothesis of depression

#### TRP

In this study, the TRP concentration was significantly lower in patients with major depression in comparison with the healthy controls (*t*: −3.93; *df* = 116, *p* < 0.001).

#### TRP breakdown to KYN due to either activated IDO1, IDO2 or TDO (KYN/TRP Ratio)

A significant positive correlation between TRP and KYN was observed only in patients with major depression, suggesting increased IDO1-, IDO2- or TDO activity only in patients with major depression, while no correlative relationship was found in the healthy controls (major depression: Spearman rank correlation coefficient: *r* = 0.253, *p* = 0.035; healthy controls: Spearman rank correlation coefficient: *r* =  − 0.005, *p* = 0.975). In addition, a trend of increased KYN/TRP ratio, reflecting TRP breakdown to KYN (due to either activated IDO1, IDO2 or TDO), was found in the patients with major depression in comparison to the healthy controls (Mann–Whitney-U: 1338.0; *p* = 0.061).

#### KYN

No significant difference in KYN concentrations was observed in the patients with major depression in comparison to the healthy controls (*t*: −0.945; *df* = 116, *p* = 0.347).

#### KA

The patients with major depression had a significantly lower concentration of neuroprotective KA in comparison to the healthy controls (Mann–Whitney-U: 1315.0; *p* = 0.046).

#### QA

No significant differences in QA concentrations and the QA/ TRP ratio were observed in the patients with majordepression in comparison to the healthy controls (QA: Mann–Whitney-U: 1376,5; *p* = 0.076; QA/TRP ratio: Mann–Whitney-U: 1641.00; *p* = 0.831).

Table 2 provides the biological assessments.

#### Body Mass Index (BMI)/Antidepressants

We examined the correlations between BDI-II scores, BMI and biological assessments using Spearman’s-Rho and partial correlation coefficients. Nearly all biological measures were significantly correlated except TRP with KYN as well as TRP and QA (see Table 3).

**Table 3:** Bivariate Rank Correlations^a^ of BDI-II scores; BMI and biological measures

	BDI-II	TRP	KYN	KYN/TRP Ratio	QA	KA
TRP	−.393^***^					
KYN	−.079	.170				
KYN/TRP Ratio	.184	−.456^***^	.741^***^			
QA	−.096	−.018	.598^***^	.573^***^		
KA	−.282^**^	.219^*^	.555^***^	.348^***^	.418^***^	
BMI	.366^***^	−.186	.278^***^	.409^***^	.327^***^	.118

**Notes.**

a Spearman’s Rho rank based on 105 Cases.

* *p* < 0.05.

** *p* < 0.01.

*** *p* < 0.001.

Controlling for BMI, the partial correlation results in Table 4 showed that only TRP and QA were not significantly correlated.

**Table 4:** Partial Correlations^a^ of biological measures controlling for body mass index (BMI).

	BDI-II	TRP	KYN	KYN/TRP Ratio	QA
TRP	−0.408^***^				
KYN	−0.239^*^	0.276^**^			
KYN/TRP Ratio	.037	−0.390^***^	0.766^***^		
QA	−0.236^*^	.019	0.662^***^	0.624^***^	
KA	−0.310^**^	0.236^*^	0.543^***^	0.391^***^	0.446^***^

**Notes.**

a Partial correlations based on 102 cases.

* *p* < 0.05.

** *p* < 0.01.

*** *p* < 0.001.

Thus, to avoid multi-collinearity problems in the multivariate analysis we selected TRP and QA representing the biological measures in this analysis.

Further, we tested for antidepressant medication effects (SSRI, SRNI, other antidepressants, and no antidepressants) and calculated Spearman’s Rho correlation coefficients for biochemical measures and BDI-II scores (see Table 5).

**Table 5:** Bivariate Rank Correlations^a^ of biological measures with BDI-II scores by types of antidepressants (*N* = 67).

	No medication	SSRI	SRNI	Other antidepressants
	*n* = 12 BDI-II	*n* = 26 BDI-II	*n* = 22 BDI-II	*n* = 8 BDI-II
TRP	−.036	−.421^*^	−.433^*^	.431
KYN	−.164	−.446^*^	.090	−.275
KYN/TRP Ratio	−.191	−.017	.383	−.515
QA	.077	−.212	.270	−.072
KA	−.473	−.141	−.241	−.587

**Notes.**

a Spearman’s Rho.

* *p* < 0.05.

** *p* < 0.01.

*** *p* < 0.001.

Patients with SSRI treatment showed a significant negative correlation between BDI-II scores and TRP concentrations as well as KYN concentrations, while patients with SNRI treatment only showed a significant negative correlation between BDI-II scores and TRP concentrations.

#### Multivariate analyses including the contributing biological and psychosocial factors

A two-step hierarchical linear regression model was used to examine potential correlates of the observed BDI-II scores. The following correlates were selected: TRP, QA, social support (SSS), Physical Functioning, Physical Role, Body Pain, Vitality (all SF-36), Occupational Requirements, Income/Financial Security, Leisure Time/Hobbies, Personality Traits, Sexuality, Friends/Acquaintances, and Housing/Living Conditions (all FLZ). To avoid issues of multi-collinearity, or due to a high number of missing values, the other SF-36 and FLZ variables have been excluded from the model. Age and sex were chosen as control variables. In our analysis, we considered sex as a categorical variable. Therefore, we measured sex with one dummy variable taking the value 1 if the patient was male and the value 0 if the patient was female.

In the first step, we regressed BDI-II scores against the control variables age and sex (Model 1). This Model 1 yields a ${R}_{\mathrm{adj.}}^{2}=0.025$; and is statistically insignificant (*p* = 0.120). In the second step, we introduced the hierarchical linear regression model predictors (Model 2). This Model 2 is highly significant and yields a ${R}_{\mathrm{adj.}}^{2}=0.860$; *p* < 0.001. Additionally, the Δ*R*^2^ = 0.838 is highly significant (*p* < 0.001), which means that the introduction of the hierarchical linear regression model predictors to the model significantly improves the explanation of the variance of BDI-II scores. In this step, the Model 2 explains 86% of the variance of the BDI-II scores and shows that low TRP concentrations, low social support (SSS), impaired Physical Role and Vitality (both SF-36), Occupational Requirements and dissatisfaction with Personality Traits (both FLZ) are significant correlates of depressive symptomatology (BDI-II scores).

Table 6 provides the psychometric Data of the SF-36, the FLZ and the SSS.

Table 7 shows the results of the Biopsychosocial Model, based on the two-step hierarchical linear regression model.

## Discussion

The clinical relevance of the inflammatory hypothesis of depression is not fully understood and subject to controversy (Wichers et al., 2005; Savitz et al., 2015; Dantzer et al., 2008). Corresponding with the inflammatory hypothesis of depression, our study results suggest a TRP breakdown (due to either activated IDOs or TDO) in patients with major depression, and these depressive patients had a barely significantly lower concentration of neuroprotective KA in comparison to the healthy controls. Contradicting the inflammatory theory of depression, the concentrations of KYN, neurotoxic QA and QA/ TRP ratio were not significantly different between depressed and healthy controls. These results suggest that there might be also minor inflammatory activation during depression in somatically healthy depressive patients that, despite the fact that TRP concentrations are significantly decreased in depressive patients, causes KYN and QA concentrations within a normal range, due to a TRP breakdown (due to either activated IDOs or TDO; KYN/TRP Ratio). Capuron et al. (2011) showed in a large study including elderly persons, that increased inflammation might be related to reduced tryptophan concentrations and increased kynurenine levels, due to induced increased tryptophan catabolism. This increased tryptophan catabolism was associated with important depressive symptoms (lassitude, reduced motivation, anorexia, and pessimism). Our present findings support the TRP-5-HT deficiency hypothesis of depression due to the deficiency of the 5-HT precursor TRP, which suggests a dysfunction of 5-HT neurotransmission during depression. Physiological changes in plasma TRP concentration influence brain 5-HT levels (Oathes, Hilt & Nitschke, 2015; Fakhoury, 2016). Thus, in an animal study, brain 5-HT concentrations were significantly elevated one hour after the rats received a dose of L-TRP (Fernstrom & Wurtman, 1971). Earlier studies reported on lower tryptophan levels rather than kynurenine changes to correlate with depression (e.g., Huang et al., 2002).

**Table 6:** Psychometric Data of the SF-36, FLZ and the SSS.

	**Patients with major depression**	Healthy controls
SF-36		
Physical Functioning^***^	Mean: 56.99 SD: 25.817 Median: 55.00 Range: 90	Mean: 96.21 SD: 8.790 Median: 100 Range: 35
Role Physical^***^	Mean: 15.07 SD: 2.697 Median: .00 Range: 100	Mean: 94.51 SD: 16.31 Median: 100 Range: 75
Pain^***^	Mean: 42.32 SD: 29.806 Median: 41.00 Range: 100	Mean: 91.71 SD: 15.523 Median: 100 Range: 59
General Health^***^	Mean: 33.04 SD: 19.007 Median: 31.25 Range: 82	Mean: 85.71 SD: 13.677 Median: 90.0 Range: 48
Vitality^***^	Mean: 23.26 SD: 17.789 Median: 20.0 Range: 85	Mean: 73.54 SD: 13.474 Median: 75.0 Range: 55
Social Functioning^***^	Mean: 30.77 SD: 24.100 Median: 25 Range: 100	Mean: 94.51 SD: 13.989 Median: 100.0 Range: 63
Role Emotional^***^	Mean: 11.60 SD: 25.427 Median: 0 Range: 100	Mean: 97.56 SD: 11.524 Median: 100 Range: 67
Mental Health^***^	Mean: 30.83 SD: 18.422 Median: 32.0 Range: 96	Mean: 86.24 SD: 9.552 Median: 88.0 Range: 52
**FLZ**		
General Health^***^	Mean: 19.15 SD: 8.894 Median: 17.0 Range: 34	Mean: 42.76 SD: 4.716 Median: 43.0 Range: 20
Occupational Requirements^***^	Mean: 30.46 SD: 11.179 Median: 30.0 Range: 42	Mean: 40.87 SD: 7.753 Median: 43.0 Range: 29
Income/Financial Security^***^	Mean: 29.37 SD: 11.675 Median: 28.0 Range: 41	Mean: 40.07 SD: 7.280 Median: 41.5 Range: 34
Leisure Time/Hobbies^***^	Mean: 30.09 SD: 10.739 Median: 30.0 Range: 42	Mean: 39.67 SD: 8.383 Median: 42.0 Range: 36
Partner Relationship^**^	Mean: 37.80 SD: 12.157 Median: 43.00 Range: 42	Mean: 44.43 SD: 5.701 Median: 46.0 Range: 19
Family Life/Children	Mean: 40.91 SD: 8.211 Median: 42.0 Range: 42	Mean: 43.70 SD: 6.342 Median: 46.00 Range: 26
Personality Traits^***^	Mean: 29.02 SD: 8.914 Median: 28.0 Range: 41	Mean: 43.15 SD: 4.181 Median: 44.00 Range: 17
Sexuality^***^	Mean: 28.98 SD: 10.269 Median: 28.0 Range: 45	Mean: 40.12 SD: 7.772 Median: 42.0 Range: 22
Friends/ Acquaintances^***^	Mean: 33.43 SD: 9.219 Median: 34.0 Range: 43	Mean: 41.85 SD: 5.931 Median: 42.0 Range: 21
Housing/Living Conditions ^***^	Mean: 40.31 SD: 7.230 Median: 42.0 Range: 30	Mean: 45.49 SD: 3.988 Median: 47.0 Range: 15
SSS^***^	Mean: 52.42 SD: 15.596 Median: 52.50 Range: 66	Mean: 69.27 SD: 7.893 Median: 72.50 Range: 25

**Notes.**

** *p* < 0.01.

*** *p* < 0.001.

**Table 7:** Biopsychosocial Model based on two-step hierarchical linear regression.^a^

Step 1 (Model 1); DV = BDI depression score	Control variables	B	Beta	S.E.	*p*	VIF
	Constant	7.771		4.926	0.118	
	Age	0.181	0.189	0.099	0.072	1.020
	Sex–male (Dummy variable)	−3.720	−0.131	2.960	0.212	1.020
		*F* = 2.167; *p* = 0.12; *R* = 0.214; *R*^2=^0.046; *R*^2^ adj. = 0.025				

Step 2 (Model 2); DV = BDI depression score	**Predictors (correlates)**	**B**	**Beta**	**S.E.**	*p*	**VIF**
	Constant	52.693		5.340	0.000	
	Age	0.015	0.016	0.045	0.742	1.485
	Sex –male (Dummy variable)	1.407	0.049	1.237	0.259	1.241
	TRP	−0.124	−0.099	0.056	**0.031**	1.332
	QA	−0.005	−0.057	0.004	0.205	1.298
	Social support (SSS)	−0.122	−0.134	0.058	**0.039**	2.648
	Physical functioning (SF-36)	−0.047	−0.098	0.036	0.197	3.728
	Physical role (SF-36)	−0.057	−0.193	0.023	**0.016**	4.016
	Pain (SF-36)	−0.044	−0.111	0.026	0.101	2.944
	Vitality (SF-36)	−0.089	−0.194	0.036	**0.016**	4.084
	Occupation/work (FLZ)	0.248	0.205	0.080	**0.003**	2.843
	Income/financial security (FLZ)	−0.017	−0.014	0.073	0.816	2.319
	Leisure time/hobbies (FLZ)	−0.009	−0.007	0.071	0.900	2.012
	Personality traits (FLZ)	−0.688	−0.511	0.110	**<0.001**	4.400
	Sexuality (FLZ)	0.035	0.027	0.074	0.634	2.184
	Friends/acquaintances (FLZ)	0.021	0.013	0.094	0.825	2.242
	Housing/living conditions (FLZ)	0.121	0.057	0.123	0.328	2.205
	*F* = 36.347; ***p ≤ 0.001***; *R* = 0.940; *R*^2=^0.884; ${R}_{\mathrm{adj.}}^{2}=0.860$				
		Δ*R*^2=^0.840; ***p ≤ 0.001***				

**Notes.**

a To avoid issues of multicollinearity General Health (SF-36), Social Functioning (SF-36), Emotional Role (SF-36), Mental Health (SF-36), and General Health (FLZ) variables have been excluded from the model. Due to a high number of missing data we have excluded Partner Relationship and Family Life/Children (both FLZ).

The results of our present study, as described above, of somatically healthy patients with major depression are partly in contrast to our previous findings on IFN-α induced depressive symptomatology in patients with chronic hepatitis C infection (Baranyi et al., 2013; Baranyi et al., 2015b). IFN-α has a weak direct influence on IDO1 as well as a stronger indirect influence via IFN-*γ* (Raison et al., 2005). Just before the outbreak of IFN-α induced depression, TRP concentrations were not significantly different to those of HCV-infected patients without IFN-α induced depressive symptomatology. Furthermore, KYN concentrations were significantly elevated in patients with IFN-α induced depression. In addition, patients with higher depression scores at six and nine months after starting of the therapy showed significantly higher levels of QA concentration. These findings strongly supported the inflammatory hypothesis as the major pathomechanism in the evolution of IFN-α induced depression (Baranyi et al., 2013; Baranyi et al., 2015b).

In a biopsychosocial model, social support is an important contributing factor (Fava et al., 2012; Baranyi et al., 2013). The multivariate analyses of this present study showed that low social support has been a correlate of higher BDI-II scores. In this study, the domains Occupational Requirements and dissatisfaction with Personality Traits in the Life Satisfaction Questionnaire (FLZ) are significant stress factors that might to contribute to a depressive symptomatology. Further significant health-related quality of life correlates of depressive symptomatology are impaired: Physical Role and impaired Vitality, both domains in SF-36. Regarding the biological aspect of a biopsychosocial model in correspondence with the univariate analyses, in this study only TRP has been a correlate of higher BDI-II scores, but not QA concentrations.

**Figure 1:**
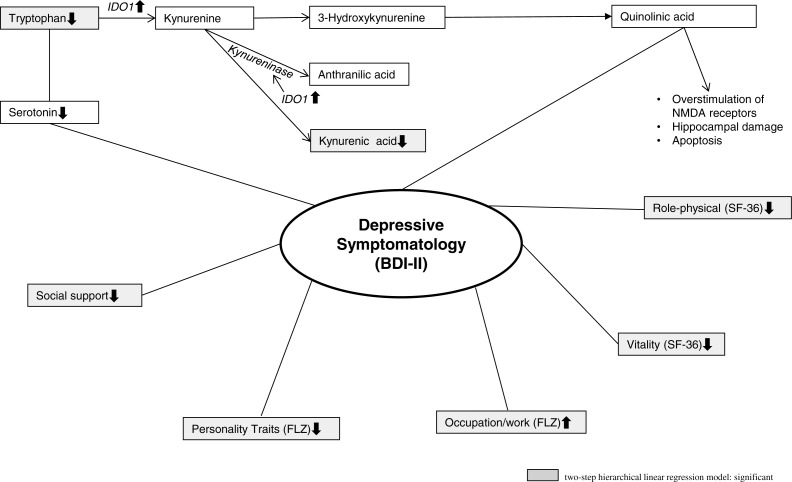
Illustrates the TRP metabolism and the biopsychosocial model of major depression.

Figure 1 shows the TRP metabolism in general and the biopsychosocial model of major depression based on the study results.

### Limitations

The results of this cross-sectional study represent important biological and psychosocial correlates of major depression. There is only correlational evidence in a cross-sectional setting which cannot decide the cause-effect relationship between symptoms and biochemical changes.

This study presents some positive correlation data for the inflammatory hypothesis (lower concentration of neuroprotective KA in depressed subjects, accelerated TRP breakdown to KYN due to either activated IDOs or TDO). However, the absence of evidence for correlation of some other inflammatory factors (e.g., KYN and QA) is not evidence for the absence or evidence against the inflammatory hypothesis of depression. Subsequent studies should also include the cytokine levels, which might impact on the study results. An increase of KYN to TRP ratio cannot serve alone as a sufficient indicator of IDO1 activity, because it indicates accelerated TRP breakdown, which could be due to either activated IDOs or TDO. Moreover, down-stream enzymes modulate KYN levels once produced. Thus, TRP may decrease due to an enhanced enzymatic breakdown without a concomitant increase of kynurenine when the product (kynurenine) is rapidly further converted by down-stream enzymes. The net effect might explain why kynurenine was not found to be increased. Antidepressant drugs, independent of their mechanism of action, are able to counter to some extent the effects of cytokines on the human brain (Dantzer et al., 2008; Hannestad, DellaGioia & Bloch, 2011). In this present study patients with SSRI treatment showed a significant negative correlation between BDI-II scores and TRP concentrations as well as KYN concentrations, while patients with SNRI treatment only showed a significant negative correlation between BDI-II scores and TRP concentrations. To study this possible effect of antidepressant medication on the measured outcome parameters, further studies should also include drug free subgroups. In general, the causative relationships have to be explored in subsequent prospective follow-up studies, and knowledge about the complex biopsychosocial pathomechanisms involved in the evolution of major depression is still limited and requires ongoing research.

## Conclusions

The inflammatory hypothesis of depression is a key hypothesis for the understanding of major depression (Dantzer et al., 2008). The results of this study and the results of our former study on IFN- *α* induced depressive symptomatology are strong evidence that major depression is not such a homogenous entity as expected. In contrast, it seems that there are different pathomechanisms involved in the development of depression. While we have found great support for the inflammatory hypothesis in depressive states with consequent immune activation (e.g., IFN-α induced depression) the impact of the inflammatory hypothesis of depression might be a little less important in somatically healthy patients with major depression and no previous immune activation. In this present study of somatically healthy patients without primarily strong inflammatory responses, depression might be the consequence of TRP deficiency and subsequent lack of 5-HT alongside biopsychosocial factors. However, inflammatory responses might be important additional pathomechanisms. Former studies have demonstrated that there exists a connection of 5-HT to the immune system via the cofactor tetrahydrobiopterin. The biosynthesis of tetrahydrobiopterin is also upregulated during immune responses (Widner et al., 2002; Murr et al., 2002).

The heterogeneous causes and types of depression might also explain the occasionally inadequate effect of SSRIs in depression and might require disease-specific individual pharmacological treatment alongside adequate psychotherapy. In conclusion, our study results argue for the validity of a biopsychosocial model with multiple pathophysiological mechanisms involved.

##  Supplemental Information

10.7717/peerj.3968/supp-1 Data S1 Dataset

## List of abbreviations

BBB: Blood-brain Barrier
BCAAs: Branched-Chain Amino Acids
BDI-II: Beck Depression Inventory
BTP: Beta-Trace Protein
FLZ: Life Satisfaction Questionnaire
HPLC: High-Performance Liquid Chromatography
IDO1: Indoleamine 2,3-dioxygenase 1
IDO2: Indoleamine 2,3-dioxygenase 2
Il-1: Interleukin-1
Il-6: Interleukin-6
IFN-*α*: Interferon-alpha
IFN-*γ*: Interferon-gamma
KA: Kynurenic Acid
KYN: Kynurenine
NMDA: N-methyl-D-aspartate
QA: Quinolinic Acid
SD: Standard Deviation
SF-36: Medical Outcome Study Form
SNRI: Serotonin–norepinephrine Re-uptake inhibitor
SSRI: Selective Serotonin Re-uptake Inhibitor
SSS: Social Support Survey
TDO: Tryptophan 2,3-Dioxygenase
TNF-*α*: Tumor Necrosis Factor-α
TRP: Tryptophan
5-HT: Serotonin
